# New Insight on the Study of the Kinetic of Biobased Polyurethanes Synthesis Based on Oleo-Chemistry

**DOI:** 10.3390/molecules24234332

**Published:** 2019-11-27

**Authors:** Julien Peyrton, Clémence Chambaretaud, Luc Avérous

**Affiliations:** 1 BioTeam/ICPEES-ECPM, UMR CNRS 7515, Université de Strasbourg, 25 rue Becquerel, CEDEX 2, 67087 Strasbourg, France; julien.peyrton@etu.unistra.fr (J.P.); clemence.chambaretaud@uha.fr (C.C.); 2 Soprema, 14 rue de Saint-Nazaire, CEDEX 1, 67025 Strasbourg, France

**Keywords:** kinetics, epoxide, ring-opening, biobased, polyurethane foam, catalyst

## Abstract

Nowadays, polyols are basic chemicals for the synthesis of a large range of polymers, such as polyurethane foams (PUF), which are produced with several other compounds, such as polyisocyanates. During the last decades, the oleo-chemistry has developed several routes from glycerides to polyols for the polyurethanes (PU) industry to replace mainly conventional fossil-based polyols. A large range of biobased polyols can be now obtained by epoxidation of the double bonds and ring-opening (RO) of the subsequent epoxides with different chemical moieties. In preliminary studies, the RO kinetics of an epoxidized model molecule (methyl oleate) with ethanol and acetic acid were investigated. Subsequently, polyols that were derived from unsaturated triglycerides were explored in the frame of e.g., PUF formulations. Different associations were studied with different mono-alcohols derived from epoxidized and ring-opened methyl oleate while using several ring-openers to model such systems and for comparison purposes. Kinetic studies were realized with the pseudo-first-order principle, meaning that hydroxyls are in large excess when compared to the isocyanate groups. The rate of isocyanate consumption was found to be dependent on the moiety located in β-position of the reactive hydroxyl, following this specific order: tertiary amine >> ether > ester. The tertiary amine in β-position of the hydroxyl tremendously increases the reactivity toward isocyanate. Consequently, a biobased reactive polyurethane catalyst was synthesized from unsaturated glycerides. These approaches offer new insights regarding the replacement of current catalysts often harmful, pungent, and volatile used in PU and PUF industry, in order to revisit this chemistry.

## 1. Introduction

The polyurethane (PU) is a very versatile family of polymer that is mainly obtained by polyaddition between polyols and polyisocyanates [1]. PUs can be used in various forms to fulfill different applications for a worldwide market of $50 Billion in 2016 due to the multiplicity of their structures. With more than 60%, foams are the largest part of this market, with segments including the furniture, bedding, insulation, building, or construction materials. Foams are elaborated through a complex formulation that is based on polyols, polyisocyanates, blowing agent, and several other additives [2,3]. Commercial foams are mainly formulated with fossil-based components. However, increasing foams are obtained from renewable resources nowadays.

The abundance and versatility of vegetable oils are the key points for replacing petrochemical products in polymer synthesis and developing very promising renewable compounds while using a well-established oleo-chemistry. During the last decades, starting from unsaturated triglycerides, extensive research [4,5,6] has been performed to obtain new macromolecular architectures. Multiple strategies were developed to obtain polyols from unsaturated glycerides for the PU industry [7] (i) the hydroformylation and ozonolysis, followed by a catalytic reduction is developed at an industrial scale, (ii) the transesterification, (iii) the introduction of hydroxyl groups via double-bonds with microorganisms is promising [8], and (iv) the epoxidation of the double bonds and subsequent the ring-opening (RO) of the epoxides. The last way keeps the initial glyceride structure, and the opportunity to synthetized different polyols structures, even at an industrial level.

In the case of vegetable oils and fats, the epoxide is mainly di-substituted and, consequently, less reactive than a terminal one. Nevertheless, several different types of reagents can be considered for the RO, such as amines [9,10,11], alcohols [12,13,14], carboxylic acids [15,16], or hydrogen halides [17,18]. The epoxidation procedure is carried out while using a short carboxylic acid [19]. Although it is not fully new, for instance, few publications are focused on the study of the RO kinetics of epoxide by acetic or formic acid [20,21].

The presently studied way to obtain PUs from poly-unsaturated triglycerides contains three steps: 1. Epoxidation of double bonds, 2. RO reaction, and 3. Polymerization with polyisocyanate. In our study, the polyunsaturated triglycerides were modeled by a fatty ester only containing one double-bond: methyl oleate. The double bond was chemically converted into epoxide by a peracetic acid that was formed in situ. The objective of this preliminary study was to understand the acid-catalyzed RO of disubstituted epoxide. To do so, a new kinetic method that was based on Nuclear magnetic resonance (NMR) was developed to monitor the epoxide RO reaction. Subsequently, it was applied to the kinetic study of acid-catalyzed RO reactions of epoxidized methyl oleate. In the second part of this paper, the reactivity of different alcohols (models) that were obtained from RO of epoxidized fatty esters with various conditions was compared in the frame of PU synthesis. The model-alcohols were synthesized by the RO of the epoxide, with acetic acid, ethanol, hydrogen halide, or diethylamine.

## 2. Materials and Methods

### 2.1. Materials

Fatty Acid Methyl Ester of Very High Oleic Sunflower Oil (FAMEVHOSO) with 3.32 mmol double bond/g was kindly supplied by the ITERG group (Canéjan, France). The FAMEVHOSO is composed of 83% of oleic acid. Table S1 presents the distribution of fatty methyl esters of unsaturated FAMEVHOSO. Glacial acetic acid (AA), toluene (99%), H_2_O_2_ 30%, ethyl acetate (99%), and ethanol (99.9%) were obtained from Fisher Scientific (Illkirch-Graffenstaden, France). Amberlyst^®^ 15H (strongly acidic cation exchanger dry), Amberlite^®^ IR120H (strongly acidic hydrogen form), CDCl_3_, phenylisocyanate (98%), dibutylamine (DBA) (99.5%), HBr (48% in water), HCl (37% in water), and diethylamine (99%) (DEA) were provided by Sigma-Aldrich (Saint-Quentin-Fallavier, France). Ethanol absolute (EtOH) was purchased from VWR (Briare, France). All of the chemicals were used without any purification.

### 2.2. Epoxidation of FAMEVHOSO

According to a previously described protocol [22], 200 g of FAMEVHOSO (0.66 mol, 1 eq), 50 g of Amberlite^®^ IR 120H (25 wt% of FAMEVHOSO) were introduced in a 1 L three-neck flask that was equipped with a reflux condenser, a magnetic stirrer, and a dropping funnel. 20 mL of acetic acid (0.35 mol, 0.5 eq) and 200 mL of toluene were added. The mixture was heated to 70 °C under vigorous magnetic stirring. Afterwards, 90 mL of H_2_O_2_ 30% (1.15 mmol, 1.7 eq) was added dropwise by the dropping funnel for 30 min. to prevent overheating and epoxide RO. The mixture was heated at 70 °C for 7 h additional hours. At the end, the mixture was recovered in 500 mL of ethyl acetate. The Amberlite^®^ IR 120H was filtered off. The organic phase was washed with saturated NaHCO_3_ solution until neutral pH. Afterwards, it was washed with brine solution, dried with anhydrous sodium sulfate, and then filtered. The solvent was evaporated under reduced pressure. The epoxidized FAMEVHSOSO (EVHOSO) was dried overnight in a vacuum oven at 40 °C. The yield was 90 mol%.

### 2.3. Ring-Opening of EVHOSO with Acetic Acid

The reaction was carried out in a round bottom flask that was equipped with a reflux condenser and a magnetic stirrer. The flask was filled with 50 g of EVHOSO 3.05 mmol epoxide/g, (0.24 mol, 1 eq) and 100 mL of acetic acid (2.8 mol, 11.5 eq). The mixture was stirred at 90 °C for 7 h. At the end, the mixture was recovered in 300 mL of ethyl acetate. The organic phase was washed with saturated NaHCO_3_ solution until neutral pH. Subsequently, it was washed with brine solution, dried with anhydrous sodium sulfate, and then filtered. The solvent was evaporated under reduced pressure. The ring-opened EVHOSO with acetic acid (EVHOSO-AA) was dried overnight in a vacuum oven at 40 °C. The yield was 82 mol%.

### 2.4. Ring-Opening of EVHOSO with Ethanol

The protocol was adapted from a previously published work [23]. The reaction was carried out in a round bottom flask that was equipped with a reflux condenser and a magnetic stirrer. The flask was filled with 50 g of EVHOSO 3.05 mmol epoxide/g, (0.15 mol, 1 eq) and 2 g of Amberlyst^®^ 15 H. 100 mL of ethanol (1.7 mol, 11.5 eq). The mixture was stirred at 70 °C for 9 h. At the end the mixture was recovered in 300 mL of ethyl acetate. The organic phase was washed with saturated NaHCO_3_ solution until neutral pH. Subsequently, it was washed with brine solution, dried with anhydrous sodium sulfate, and then filtered. The solvent was evaporated under reduced pressure. The ring-opened EVHOSO with ethanol (EVHOSO-EtOH) was dried overnight in a vacuum oven at 40 °C. The yield was 92 mol%.

### 2.5. Ring-Opening of EVHOSO with Diethylamine

The protocol was adapted from a previously published work [24]. The reaction was carried out in a round bottom flask that was equipped with a reflux condenser and a magnetic stirrer. The flask was filled with 5 g of anhydrous ZnCl_2_ (0.5 eq). 25 g of EVHOSO 3.05 mmol epoxide/g (76 mmol, 1 eq) was dissolved in 20 mL of diethylamine (193 mmol, 2.5 eq). The solution was added in the flask. The mixture was stirred at reflux for 15 h. At the end, the mixture was recovered in 300 mL of ethyl acetate and deionized water. The organic phase was washed with saturated NaHCO_3_ solution until neutral pH. Afterwards, it was washed with brine solution, dried with anhydrous sodium sulfate, and then filtered. The solvent was evaporated under reduced pressure. The ring-opened EVHOSO with diethylamine (EVHOSO-DEA) was dried overnight in a vacuum oven at 40 °C. The yield was 90 mol%.

### 2.6. Ring-Opening of EVHOSO with Different Hydrogen Halides

The reaction was carried out in a round bottom flask that was equipped with a reflux condenser and a magnetic stirrer. The flask was filled with 25 g of EVHOSO 3.05 mmol epoxide/g (76 mmol, 1 eq) dissolved in 15 mL of acetone. The halogen halide (1.5 eq) was added dropwise for 15 min. to avoid overheating. The mixture was stirred at room temperature for 30 min. At the end, the mixture was recovered in 300 mL of ethyl acetate and deionized water. The organic phase was washed with saturated NaHCO_3_ solution until neutral pH. Subsequently, it was washed with brine solution, dried over anhydrous sodium sulfate, and then filtered. The solvent was evaporated under reduced pressure. The ring-opened EVHOSO with hydrochloric acid (EVHOSO-HCl) or hydrobromic acid (EVHOSO-HBr) was dried overnight in a vacuum oven at 40 °C. The yield was 99 mol%.

### 2.7. Kinetic Study of Epoxide Ring Opening with Ethanol

In a typical procedure, 0.5 g of EVHOSO (1.7 mmol, 1 eq) and between 4 to 20 wt% (depending on the experiment) of Amberlyst^®^ 15H in a 50 mL round bottom flask was heated to the desired temperature while using a hot plate that was equipped with magnetic stirring. When the temperature was attained, 3.8 mL of absolute ethanol was added (65 mmol, 38 eq). A few drops of the reaction mixture were taken at different reaction times and recovered in ethyl acetate and washed two times with water to remove any trace of acid. The solvent was evaporated on a rotary evaporator. The drops of oil were recovered with CDCl_3_ and analyzed by NMR.

### 2.8. Kinetic Study of Urethane Formation

In a typical procedure, 3 g of VHOSO-AA (7.2 mmol, 11.5 eq) was introduced in a round bottom flask that was equipped with a magnetic stirrer. The flask was heated to the desired temperature. The flask was put under vacuum for 15 min. and then flushed with argon. 1 mL of a 0.62 mol/L solution of phenyl isocyanate in toluene was introduced. The conditions were set to avoid any contact with humidity or another nucleophile. The isocyanate content ([NCO]) over time was determined by taking aliquots of 0.4 mL quenched by a DBA solution. [NCO] was determined by potential titration of the excess of DBA by an acid.

### 2.9. Kinetic Model of Ring-Opening

#### 2.9.1. Mechanism of Ring-Opening

**Scheme 1:**
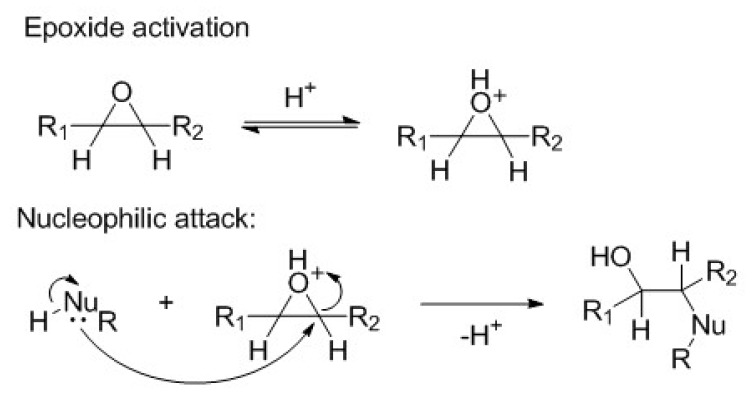
General mechanism of an acidic catalyzed nucleophile ring-opening (RO).

The RO of the epoxide is catalyzed by acid [25], as shown in Scheme 1. The nucleophilic attack is favored by the enhancement of the electrophilic character of epoxide group carbons. The nucleophilic species can be alcohol, carboxylic acid, water, hydrogen halide, etc.

The reaction forms a secondary hydroxyl and another group, depending on the nature of the nucleophile. Reactions with acid groups, such as carboxylic acid or halogen halide, are self-catalyzed.

#### 2.9.2. Kinetic Equations

The activation step that is presented in Scheme 1 is considered to be fast. Subsequently, the nucleophilic attack is the rate-determining step. The acid catalyzed RO rate is described by Equation (1):

(1) $$r=k*{\left[Ep\right]}^{\alpha }*{\left[B\right]}^{\beta }*{\left[Cat\right]}^{\delta }$$

where *k* is the reaction rate constant, [*Ep*] is the epoxide concentration, [*B*] the RO reagent concentration, [*Cat*] the catalyst concentration, and *α*, *β* and *δ* are the respective partial orders. The consummation of epoxide over time, depending on the reaction rate, is written as Equation (2):

(2) $$-\frac{d\left[Ep\right]}{dt}=k*{\left[Ep\right]}^{\alpha }*{\left[B\right]}^{\beta }*{\left[Cat\right]}^{\delta }$$

The kinetic parameters were determined while using a pseudo-first order assumption [26]. The nucleophile (*B*) was in large excess as compared to the epoxide groups (*Ep*). Subsequently, the concentration of the nucleophile ([*B*]) was considered to be constant during the reaction. Furthermore, the catalyst is regenerated along the reaction, [*Cat*] is a constant over time. When considering these hypotheses, the rate of reaction that is expressed in Equation (2) is transformed in Equation (3):

(3) $$-d\frac{\left[Ep\right]}{dt}=k_{app}*{\left[Ep\right]}^{\alpha }$$

where *k_app_* is a constant, as expressed in Equation (4):

(4) $$k_{app}=k*{\left[B\right]}^{\beta }*{\left[Cat\right]}^{\delta }$$

### 2.10. NMR

The NMR analyses were realized on a 400 MHz Bruker spectrometer. The ^1^H number of scans was set to 32. Each spectrum was calibrated with the CDCl_3_ signals, being set at 7.26 ppm.

### 2.11. NCO Concentration Measurement

The isocyanate content [*NCO*] was determined by the adaptation of ISO 14896:2009. Aliquots were dissolved in a 20 mL solution of 5 × 10^−3^ mol/L dibutylamine in toluene. The resulting mixture was stirred for 20 min. Afterwards, 20 mL of acetone was added to avoid a dephasing of the solution and the excess of dibutylamine was titrated with an automatic titrator by a 4.6 × 10^−3^ mol/L molar solution of HCl. The equivalence was determined by a potential leap. [*NCO*] was calculated with Equation (5):

(5) $$\left[NCO\right]=\frac{((V_{Bl}-V_{eq})*\left[HCl\right]}{V_{Aliquot}}$$

with *V_Bl_* the equivalence volume of 20 mL of dibutylamine solution, *V_eq_* the equivalence volume of the aliquot, [*HCl*] the chlorhydric acid concentration, and *V_aliquot_* the volume of solution taken from the solution for each kinetic measurement.

## 3. Results

### 3.1. Synthesis of EVHOSO

Table S1 presents FAMEVHOSO data. The average double bond per molecule is 1 by calculation. It is well known that unsaturated fatty acids are sensitive to UV oxidation [27]. An NMR measurement was undertaken to control the double bond quantity before the epoxidation. 0.93 double bonds per molecule were calculated by the integration of the proton of the double bond on the NMR spectrum (Figure S1). The FAMEVHOSO was in-situ epoxidized with peracid in a biphasic system. The reaction converts 90 % of the double bonds in epoxides (Figure S2). Side reactions, such as RO by acetic acid, limit the reaction yield. The number of epoxides per molecule is only 0.83.

### 3.2. Kinetics of Epoxide Ring-Opening by Ethanol

The NMR method that is described in Appendix A.1 on the RO with acetic acid was applied to the RO with ethanol.

#### 3.2.1. Determination of the Epoxide Partial Order

**Figure 1:**
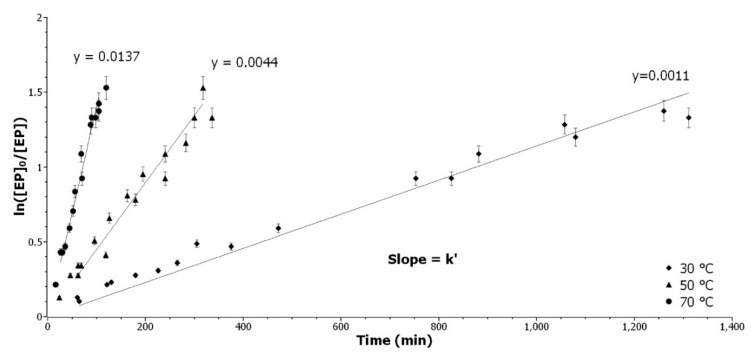
Determination of the epoxide partial order. Representation of ln([Ep]_0_/[Ep]) as a function of time at 30 °C (◆), 50 °C (▲), and 70 °C (●).

The epoxide partial order was determined at three temperatures, with all other parameters remaining equal. The pseudo-first order was applied by introducing a large excess (11 eq) of ethanol and constant catalyst content. Integrating Equation (3) with α = 1 gives Equation (6):

(6) $$\ln(\frac{{\left[Ep\right]}_{0}}{\left[Ep\right]})=\ln(\frac{1}{1-\chi })=k_{app\_EtOH}*t$$

where *χ* is the yield of the reaction, *t* the time in minute, and *k_app_EtOH_* the pseudo-reaction rate constant. ln[*Ep*]_0_/[*Ep*] was presented as a function of time in Figure 1.

The model of a partial order of 1 for the epoxide is well confirmed by experiments at 30, 50, and 70 °C, respectively. The range of validity of the method is between 20–95 % of conversion due to the exponential character of the conversion against time, as demonstrated with the acetic acid kinetic experiment (Appendix A.2).

#### 3.2.2. Determination of the Catalyst Partial Order

**Figure 2:**
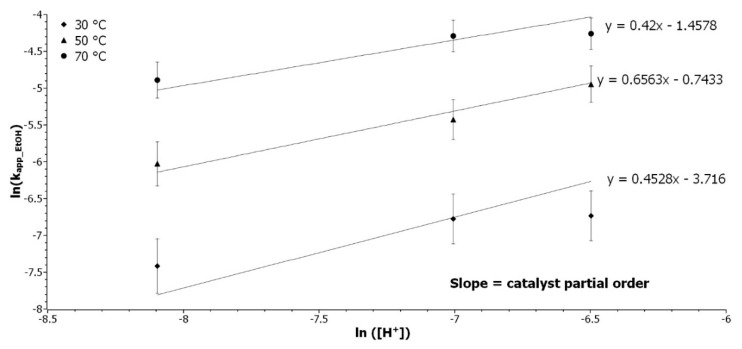
Determination of the catalyst partial order. Representation of ln(k_app_EtOH_) as a function of ln(H^+^) at 30 (◆), 50 (▲), and 70 °C (●).

Experiments with catalyst content variations from 4 to 20 wt% were performed at 30, 50, and 70 °C to determine the partial order of [Cat]. k_app_EtOH_ were determined by the linear regression of ln([Ep]_0_/[Ep]) as a function of time, and Table S2 presents the results. The catalyst is an acidic resin of divinylbenzene and styrene sulfonated [28]. The correlation between [*H*^+^] and the mass of catalyst was determined by the pH measurement of 49 mg to 1 g of resin in 20 mL of water (Figure S3). Equation (7) expresses the application of Equation (4) in the ethanol RO:

(7) $$k_{app\_EtOH}=k_{EtOH}*{\left[EtOH\right]}^{\beta }*{\left[H^{+}\right]}^{\delta }$$

where [*EtOH*] and [*H*^+^] are the concentration of ethanol and acid, respectively. The partial order of catalyst was determined by the slope of the linear regression of ln(*k_app_EtOH_*) as a function of ln([Cat]), as presented in Figure 2.

The coefficients of determination are between 0.93 and 0.98 and the three slopes are tending toward 0.5, which represent the catalyst partial order. It was expected that the catalyst would have a higher influence on the reaction rate. The catalyst is decreasing the activation energy in the thermodynamic side of the reaction, but it has less influence on the kinetics. The right amount of catalyst can be selected, depending on the thermal sensibility of the epoxidized studied molecule.

#### 3.2.3. Determination of the Ethanol Partial Order

**Figure 3:**
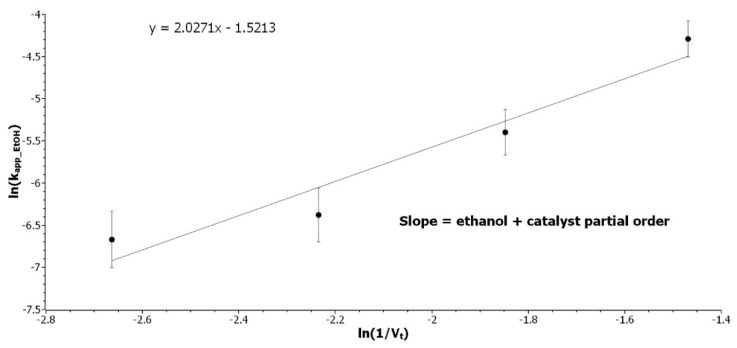
Determination of the sum of the ethanol and catalyst partial order. Representation of ln(k_app_EtOH_) as a function of ln(1/V_t_) at 70 °C with 12 wt% catalyst loading.

Experiments with [EtOH] variations from 15 to 4.5 mol/L were performed at 70 °C to determine the partial order of [EtOH]. The catalyst loading was kept constant then, with the volume variation, [*H*^+^] was varying along experiments. *k_app_EtOH_*, as expressed in Equation (7), can be expressed as a function of the total volume of solution, giving Equation (8):

(8) $$k_{app\_EtOH}=k_{EtOH}*{\left(\frac{V_{EtOH}*d_{EtOH}}{V_{t}}\right)}^{\beta }*{\left(\frac{m_{cat}*IA_{eq}}{V_{t}}\right)}^{\delta }$$

where *V_EtOH_* is the volume of ethanol introduced in the solution, *m_cat_*, the mass of catalyst, and *IA_eq_* is the acid equivalent of the resin determined by Figure S3. By logarithmic transformation and rearrangement, ln(*k_app_EtOH_*) is expressed in Equation (9) as a linear function of ln(1/*V_t_*), with the sum of partial order as the slope.

(9) $$\ln(k_{app\_EtOH})=K+(\beta +\delta )*\ln\left(\frac{1}{V_{t}}\right)$$

where *K* is a constant, including *V_EtOH_*, *d_EtOH_*, *m_cat_*, *IA_Eq_*, and *k_EtOH_*. Figure 3 represents the experimental results.

The sum of the partial order of [*H*^+^] and [*EtOH*] is 2, as the partial order of [*H*^+^] was determined to be 0.5, the partial order with respect to [*EtOH*] is 1.5. These results were confirmed by an experiment design to compensate for the dilution of catalyst that was induced by the change of concentration. Equation (10) describes the final equation rate:

(10) $$r=k_{EtOH}*{\left[Ep\right]}^{1}*{\left[EtOH\right]}^{1.5}{\left[H^{+}\right]}^{0.5}$$

The major factor impacting the reaction rate is the [*EtOH*], followed by the [*Ep*], and then the [*H*^+^]. This order is only accurate if the catalyst is present; otherwise, the reaction is slower. In this case, there is no activation step when compared to the mechanism presented in Scheme 1.

#### 3.2.4. Thermodynamics Data

Once the reaction rate determined, the thermodynamic constants were calculated and are summarized in Table 1. The sum of the catalyst partial order and the ethanol is the same as the partial order of [*AA*]. It can also confirm hypotheses that are given in the literature [20,21] regarding the autocatalysis of the acetic acid due to its acidic properties. The separation between the catalysis and the reagent action in the acetic acid case would be possible by the isolation of the reaction intermediate. Overall, the order of the reaction is the same for both reactions: third order.

The rate constant at 70 °C, *k* (70 °C), is an indication of the rate of a chemical reaction at this specified temperature [29]. The corresponding unit depends on the reaction order. From a kinetic point of view, the ethanol RO is faster when compared to the case with acetic acid due to the two decades difference in terms of *k* (70 °C). The reaction constant is a function of the temperature following an Arrhenius law, as described by Equation (11):

(11) $$k\left(T\right)=A*\exp\left(\frac{E_{A}}{R*T}\right)$$

where *A* is the frequency factor, E_a_ the activation energy of the reaction, *R* the gas constant, and *T* the temperature in Kelvin. E_a_ is determined by the slope of the linear regression of ln(*k*) as a function of the inverse of the temperature (Figure S4). It represents the energy barrier to overcome to form the intermediate state. E_a_ can be decreased while using a catalyst, but the catalyst loading does not affect it (Figure S4). The EVHOSO-EtOH intermediate state requires less energy to be formed than EVHOSO-AA. The enthalpy of activation (*ΔH^‡^*) is the energy difference between the reagents and the intermediate state [30]. It was calculated according to Equation (12)

(12) $$∆H^{\neq }=E_{A}-RT$$

An exothermic reaction has a negative Δ*H^‡^* and both intermediate RO reactions are endothermic. They absorb energy from the environment to reach an intermediate state. The entropy of activation variation (Δ*S^‡^*) is a measurement of the disorders of the reaction. Negative Δ*S^‡^* represents the loss of freedom or an order increase [30]. Kinetics data Δ*S^‡^* is calculated via Equation (13):

(13) $$∆S^{\neq }=R*\left[\ln\left(\frac{h*k_{b}}{k*T}\right)+\frac{∆H^{\neq }}{RT}\right]$$

where *h* is the Planck constant, *k* the Boltzman constant, *k_b_* the rate constant, *T* the temperature, and *R* the gas constant. In RO, by acetic acid or ethanol, the intermediate states have fewer degrees of freedom than the reactants. The entropy variation is more important in a previous publication [20], because the model molecule studied is based on epoxidized soybean oil with more than one epoxide. The loss of freedom degree is then higher and can explain the gaps in Δ*S^‡^* and E_a_.

The sign of the free enthalpy of activation Δ*G^‡^* is the combination of the entropic and enthalpic contribution, representing the spontaneity of the reaction (Equation (14)).

(14) $$∆G^{\neq }=∆H^{\neq }-T∆S^{\neq }$$

A negative Δ*G^‡^* indicates a spontaneous reaction [30]. From the thermodynamic point of view, both RO intermediates are not spontaneously formed. Thermodynamics is then not favorable to reach the intermediate of the RO of epoxide with acetic acid or ethanol, but the temperature and the kinetic are making the reaction possible in less than 10 h.

### 3.3. Kinetic Study of Urethane Formation from Fatty Acid

The kinetics of the reaction between an isocyanate and an alcohol derived from a fatty acid was investigated. The phenyl isocyanate was chosen to model the methylene diphenyl diisocyanate (MDI) that was used in foaming processes. EVHOSO-AA was the alcohol used for this study. The measurements were made while using the pseudo first order principle [30] by putting the hydroxyl moieties in large excess and measuring the concentration of isocyanate [*NCO*] over time. The same model as for the RO kinetic can be applied. The reaction proceeds without catalyst, and Equations (15) and (16) are then expressed.

(15) $$-d\frac{\left[NCO\right]}{dt}=k_{app\_U}*{\left[NCO\right]}^{\alpha }$$

where α is the partial order of the isocyanate concentration ([*NCO*]) and *k_app_U_* is:

(16) $$k_{app\_U}=k*{\left[OH\right]}^{\beta }$$

The determination of isocyanate content method was tested with known phenylisocyanate contents before starting the kinetics measurement (Figure S5). The precision of the method was around 96% and the studied concentration range was from 7 × 10^−3^ to 0.6 mol/L.

#### 3.3.1. Determination of the Isocyanate Partial Order

**Figure 4:**
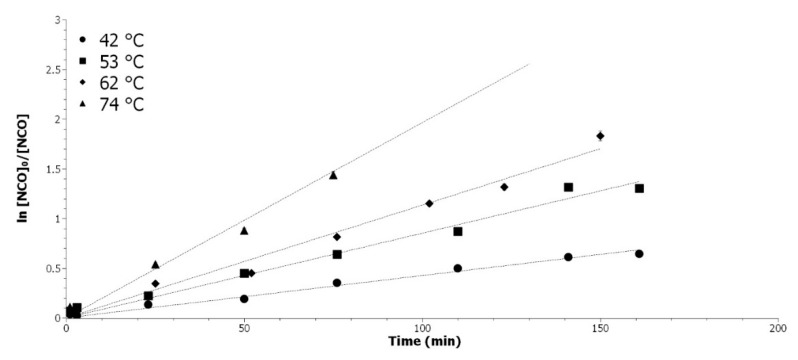
Determination of the [NCO] partial order. Variation of ln([NCO]_0_/[NCO]) in function of time at 42 °C (●), 53 °C (■), 62 °C (◆), and 74 °C (▲).

The isocyanate partial order was determined by variation of the temperature, with all other parameters being equal. The pseudo first order was applied by introducing a large excess (11 eq) of VHOSO-AA. While considering Equation (15), the logarithm of [*NCO*]_0_/[*NCO*] was represented as a function of time on Figure 4.

The partial order of 1 for the isocyanate is well confirmed by experiments at different temperatures, which are 42, 53, 62, and 74 °C. For the conversion above 99 %, the limit of the titration techniques was attained. The back titration of the [NCO] is determined by Equation (5) and a 1% difference between V_eq_ and V_bl_ is the limit of detection of the titration method.

#### 3.3.2. Determination of the Hydroxyl Partial Order

**Figure 5:**
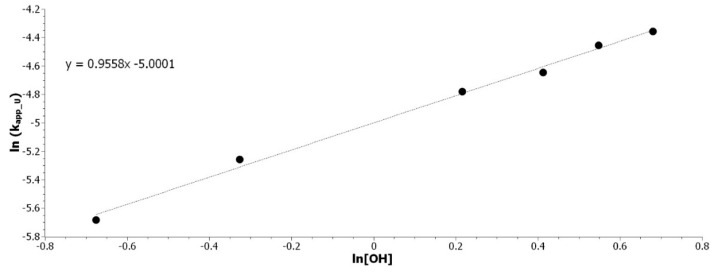
Determination of hydroxyl partial order. Linear regression of ln(k_app_U_) as a function of ln([OH]).

The determination of the hydroxyl partial order is made by varying the concentration of hydroxyl, while maintaining the great excess as compared to the isocyanate group. The variation of concentration was made by diluting the solution with toluene. The determination of k_app_U_ with 0.51, 0.72, 1.24, 1.51, 1.73, and 1.98 mol/L of hydroxyl are, respectively, presented in Figure S6. The slope of the linear regression of ln(k_app_U_) in function of ln([OH]) that is represented in Figure 5 is able to establish the partial order of hydroxyl.

The coefficient of determination is 0.995. The partial order of [OH] can be round up to 1. Table 2 presents the final rate equation with the thermodynamic data of the intermediate state. The partial order of hydroxyl and isocyanate were both equal to 1. This result is in perfect agreement with the results that were found in the literature [31,32].

The energy of activation (44 kJ/mol) is in good agreement with the results from the literature. The energy of activation reported for a reaction between phenyl isocyanate and 2-butanol is 41 kJ/mol [32] and 52 kJ/mol [33] for a reaction with or without xylene as solvent, respectively. The reaction rate constant that is presented in Table 2 (1.0 × 10^−3^ L mol^−1^ min^−1^) is six times smaller than data from the literature [32] for the reaction between secondary alcohol and phenylisocyanate. The 2-butanol is less sterically hindered than the mono-alcohols that were derived from methyl oleate. For instance, the reaction rate constant ratio between 2-hexanol and 3-hexanol was recently proved to be 1.5 for a reaction with an isocyanate [34].

From the value of the activation energy and the reaction constant of 6.0 × 10^−3^ L mol^−1^ min^−1^ enthalpy, entropy, and free energy were calculated with Equations (12)–(14), respectively. The large negative entropy of activation is an indication of the dissociation of the charged centers in the activation complex [33]. The difference of free energy of activation can be explained by better stabilization of the intermediate complex by toluene when compared to xylene [35].

The rate constant is similar to the one that was found for EVHOSO-EtOH with 20 °C of difference. By applying the Arrhenius Equation (11), the reaction rate at 70 °C for urethane formation is 1 × 10^−2^ L mol^−1^ min^−1^. The reaction between hydroxyl and aromatic isocyanate is faster than the RO by ethanol of a di-substituted epoxide. The urethane formation is not catalyzed. Catalysts, such as tertiary amine or tin salt, substantially reduce the E_a_, which increases the reaction rate by several orders of magnitude. The frequency factor makes the rate of the RO with ethanol faster with temperature, despite the difference of activation energies (Figure S7). The ΔS^‡^ of the urethane formation is higher than the one of RO with ethanol. The transition state has fewer degrees of freedom in urethane formation.

The kinetics and thermodynamics parameters of the reaction between a fatty ester alcohol and an aromatic isocyanate were determined. These parameters are specific to the studied reaction and they cannot be generalized to all isocyanates and hydroxyl substrates.

### 3.4. Study of the Reactivity with Different Isocyanates Structures

This section aims to compare the reactivity of different isocyanate and fatty ester alcohol, since different chemical architectures are available for polyols and/or polyisocyanates to produce a broad range of materials for the polyurethane industry. The reactivity of the system is an important factor, especially for foam, where the polyaddition must be fast.

**Figure 6:**
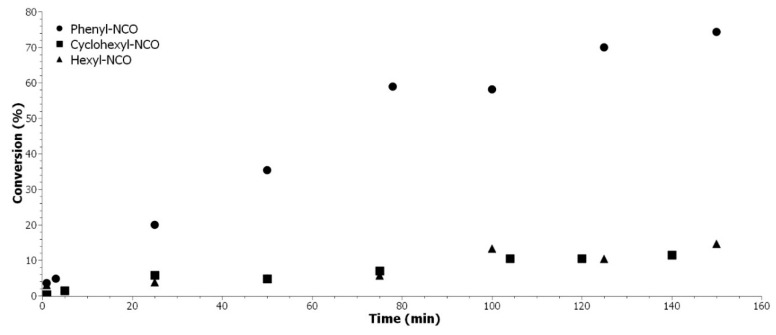
Reactivity comparison in urethane formation with EVHOSO-AA of different isocyanate structure: aromatic (●), aliphatic (▲), and cyclo-aliphatic (■).

Different isocyanate structures were compared in terms of reactivity with our model EVHOSO-AA: aromatic, aliphatic, and cycloaliphatic. The results are presented in Figure 6 once again demonstrated the higher reactivity of the aromatic isocyanate on agreement with previous results [36,37].

**Figure 7:**
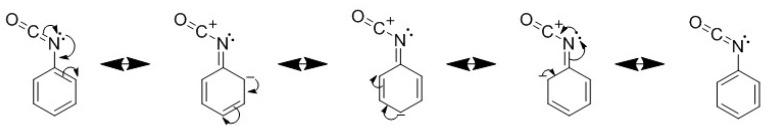
Tautomer conformation of phenyl isocyanate.

The reactions from Figure 6 were performed in the same conditions with an excess of hydroxyl moiety. Both aliphatic isocyanates have the same reactivity toward hydroxyl. The steric hindrance due to the cycle (cyclo-aliphatic chemical) does not influence the beginning of the reaction (less than 15% of conversion) with a secondary alcohol. The higher reactivity of aromatic isocyanate is due to the tautomer conformation that is presented in Figure 7.

The delocalization of the lone pair of electrons of the nitrogen atom on the aromatic cycle is increasing the electrophile character of the carbon [36,37]. It can be easily attacked by nucleophile like hydroxyl moieties. Our model that is based on fatty ester hydroxyl confirms this trend. For foams, the necessary fast polyaddition can be reached by the use of p-MDI, due to its aromatic character, which also leads to higher mechanical properties. Our model reaction confirms that, despite the steric hindrance of the aromatic moieties and the secondary character of the corresponding hydroxyl, aromatic isocyanates are the most suitable chemical for foam formulation with hydroxyl that is derived from fatty esters.

### 3.5. Synthesis of a Mono-Alcohol Model from Epoxidized Fatty Ester

**Figure 8:**
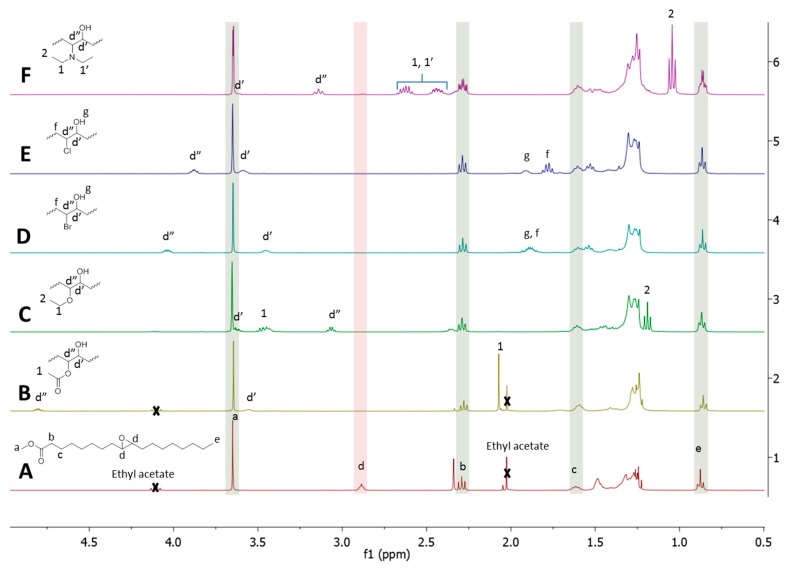
NMR Spectrum of the initial epoxidized fatty methyl ester (**A**) and after the RO with acetic acid (**B**), ethanol (**C**), hydrobromic acid (**D**), hydrochloric acid (**E**), and diethylamine (**F**).

RO can be conducted with alcohol, carboxylic acid, hydrogen halide, and secondary amine leading, respectively, to ether, ester, halide, and tertiary amine groups plus a secondary hydroxyl group. A series of different alcohols that were derived from fatty ester were synthesized by epoxide RO, with ethanol, acetic acid, HCl, HBr, and DEA (Figure 8).

There is a clear disappearance of the epoxide signal (d) located at 2.8 ppm on the NMR spectrum for all the RO reagents. Signals that are characteristic of the proton located in alpha position to the newly created hydroxyl (d’) are located in the 3.2–3.7 ppm zone. The signal located at 3.7 ppm, which is characteristic of the methyl close to the fatty ester bond (a), is constant in all products indicating non-significant ester breaking by transesterification, hydrolysis, or amidation. The RO protocols that were developed with the EVHOSO model molecule are efficient and they cause no significant ester bond breaking. These characteristics are important for the RO of more complex oils, such as triglycerides bearing several epoxide groups.

### 3.6. Evaluation of the Potential from Alcohols Derived from Fatty Acid in Polyurethane Application

**Figure 9:**
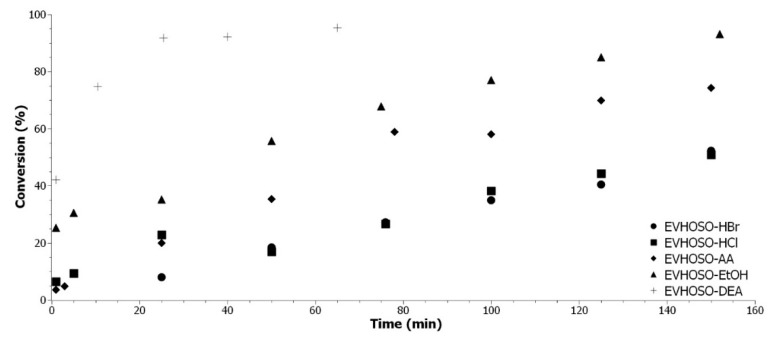
Reactivity comparison in urethane formation with phenyl isocyanate with different RO FAMEVHOSO: EVHOSO-HBr (●), EVHOSO-HCl (■), EVHOSO-AA (◆), EVHOSO-EtOH (▲), and EVHOSO-DEA (+).

The reactivity toward isocyanate of different models based on hydroxyl groups was investigated. The potential of the previously synthesized model alcohol in a urethane material was investigated through the scope of the reactivity. The reactions were carried out with a constant concentration of hydroxyl and isocyanate. The phenyl isocyanate concentration was followed over time by taking aliquots of the reaction. The results that are presented in Figure 9 show a clear tendency of reactivity with this evolution from the lowest to the highest, EVHOSO-HCl/HBr < EVHOSO-AA < EVHOSO-EtOH << EVHOSO-DEA.

**Figure 10:**
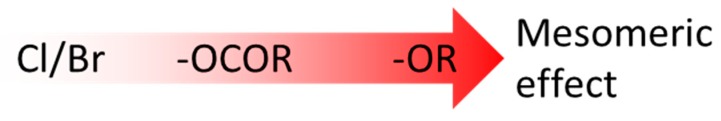
Electron releasing effect by mesomeric effect.

As clearly demonstrated, the aromatic isocyanates are very reactive toward nucleophilic attack. The surrounding of the active hydrogen group impacts the reactivity. The electron releasing groups increase the electron density of the hydroxyl by mesomeric or inductive effect. The studied groups have an inductive withdrawing effect. Except for tertiary amine, they are all electron releasing groups by mesomeric effect [38]. In general, the mesomeric effect is predominant. Furthermore, the inductive effect is decreasing with distance [39]. In our case, the two carbons distance between the inductive electron withdrawing group and the hydroxyl decreased the effect. The reactivity is then explained by the difference in strength of electron releasing groups according to the evolution that is presented in Figure 10. The EVHOSO-EtOH has a richer electron density around the hydroxyl group when compared to EVHOSO-AA and EVHOSO-Cl/Br.

When considering the tertiary amine, the inductive effect reduced by the two carbons distance is counterbalanced by a catalytic effect. In the polyurethane industry, the catalytic activity of the amine by complexation with the isocyanate or by alcohol deprotonation is well established [40,41,42]. Afterwards, the experiments confirmed the catalytic activity of the EVHOSO-DEA with aromatic or aliphatic isocyanate (Figure S8). The proximity of the reactive hydroxyl and tertiary amine must increase the corresponding catalytic activity.

## 4. Conclusions

This paper leads to different main and new results while considering the literature. An innovative NMR method was developed and successfully applied to determine the complete kinetic parameters of the EVHOSO RO reaction with ethanol. The calculated activation energy was determined, with 54 and 63 kJ/mol for the RO reaction with ethanol and acetic acid, respectively. This study led to a better understanding of the acid catalyzed RO reaction of epoxides.

Model-alcohols were successfully synthetized by epoxide RO reaction with acetic acid, ethanol, hydrogen halide, and diethylamine. The reactivity comparison with the phenyl isocyanate shows a clear tendency, with a clear evolution from the lowest to the highest, EVHOSO-HCl/HBr < EVHOSO-AA < EVHOSO-EtOH << EVHOSO-DEA.

This work can be considered as a model to better understand the oleo-chemistry approaches leading to biobased polyols. In this frame, the RO of multi-epoxidized oil with hydrogen halide, ethanol and acetic acid, or diethylamine is leading to biobased reactive additives, polyols or catalysts, respectively. The transition from model to practical application is often complex due to the limitations of the model. However, these approaches offer new insights on the replacement of current catalysts, additives, and polyols often fossil-based, used in the PUF industry, in order to revisit this chemistry. In the future, the accuracy of the kinetic model for the RO reaction needs to be tested on more complex oils. The reactivity of future triglyceride based polyols could be adjusted, depending on the RO reagent used. The potential of each RO reaction to provide a biobased substitute for the actual additives or polyols in the PUF industry could be investigated.

## Figures and Tables

**Table 1:** Thermodynamics data of the epoxidized Fatty Acid Methyl Ester of Very High Oleic Sunflower Oil (EVHOSO) ring-opening reaction with acetic acid and ethanol.

Thermodynamics Data	Acetic Acid [19] ^1^	Acetic Acid	Ethanol
Equation rate	$r=k_{AA}*{\left[Ep\right]}^{1}*{\left[AA\right]}^{2}$		$r=k_{EtOH}*{\left[Ep\right]}^{1}*{\left[EtOH\right]}^{1.5}*{\left[H^{+}\right]}^{0.5}$
k (70 °C) (L^2^·mol^−2^·min^−1^)	3.3 × 10^−5^	(2 ± 0.2) × 10^−5^	(7.8 ± 0.4) × 10^−3^
Frequency factor (min)	2.31 × 10^7^	(8.8 ± 0.8) × 10^4^	(1.28 ± 0.06) × 10^6^
E_a_ (kJ/mol)	66	63 ± 6	54 ± 3
ΔH^‡^ (70 °C) (kJ/mol)	63	60 ± 5	51 ± 3
ΔS^‡^ (70 °C) (J/mol/K)	−182	−160 ± 10	−138 ± 7
ΔG^‡^ (70 °C) (kJ/mol)	126	120 ± 10	98 ± 5

^1^ RO kinetic study made on epoxidized soybean oil by measuring the epoxide function concentration.

**Table 2:** Thermodynamics data of urethane formation by the reaction between phenyl isocyanate and EVHOSO with acetic acid (EVHOSO-AA).

Thermodynamics Data	Ref [32] ^1^	PIC ^2^/EVHOSO-AA
Equation rate	$r=k_{AA}*{\left[OH\right]}^{1}*{\left[NCO\right]}^{1}$	
k (25 °C) (L mol^−1^ min^−1^)	6.0 × 10^−3^	(1.0 ± 0.05) × 10^−3^
Frequency factor (min)		(5.3 ± 0.3) × 10^4^
E_a_ (kJ/mol)	41	44 ± 2
ΔH^‡^ (50 °C) (kJ/mol)	39	41 ± 2
ΔS^‡^ (50 °C) (J/mol/K)	−198	−160 ± 8
ΔG^‡^ (50 °C) (kJ/mol)	103	92 ± 5

^1^ Reaction between 2-propanol and phenylisocyanate in xylene, ^2^ Phenylisocyanate.

**Figure A1:**
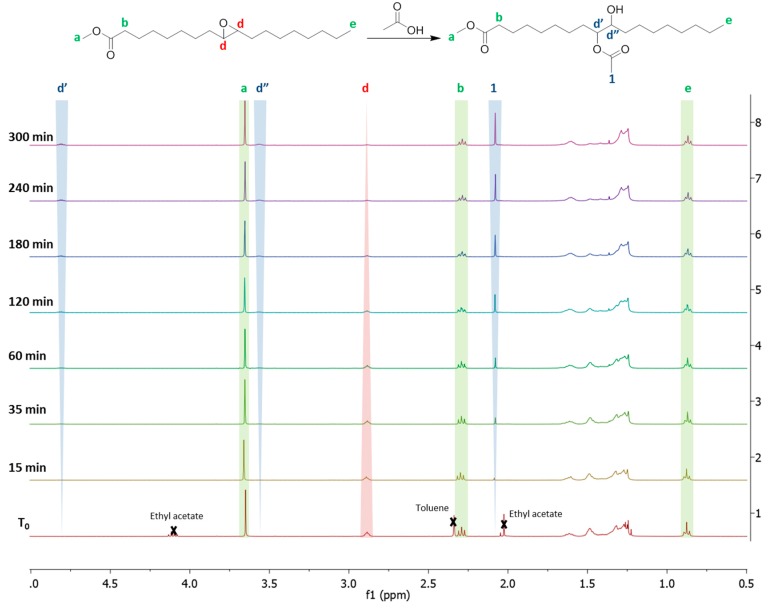
NMR analysis of epoxidized FAMEVHOSO (T_0_) and the oil after **15**, **35**, **60**, **120**, **180**, **240** and 300 min. of reaction at 90 °C with acetic acid.

**Figure A2:**
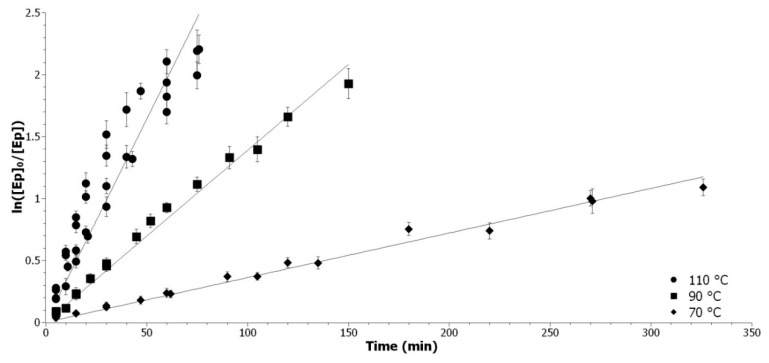
Determination of the epoxide partial order. Representation of ln([Ep]/[Ep]_0_) as a function of time at 70 °C (◆), 90 °C (▲) and 110 °C (●).

**Figure A3:**
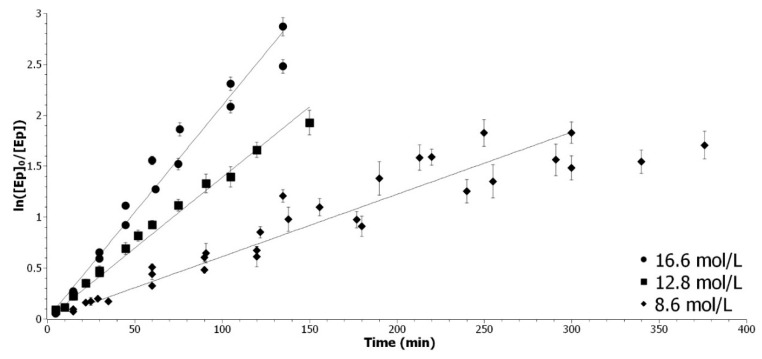
Determination of the k_app_AA_ with 8.6 mol/L (◆), 12.8 mol/L (▲) and 16.6 mol/L (●) of acetic acid.

**Figure A4:**
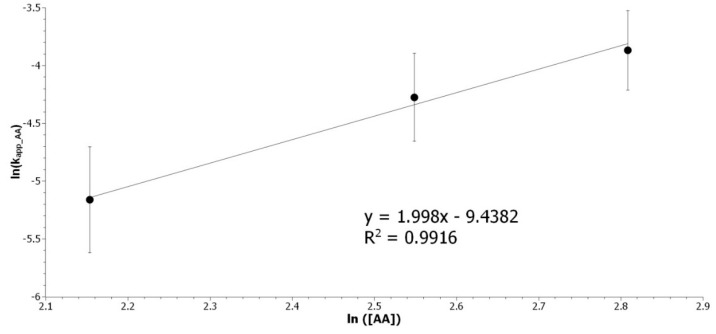
Determination of the acetic acid partial order. Representation of ln(k_app_AA_) as a function of ln ([AA]).

**Figure A5:**
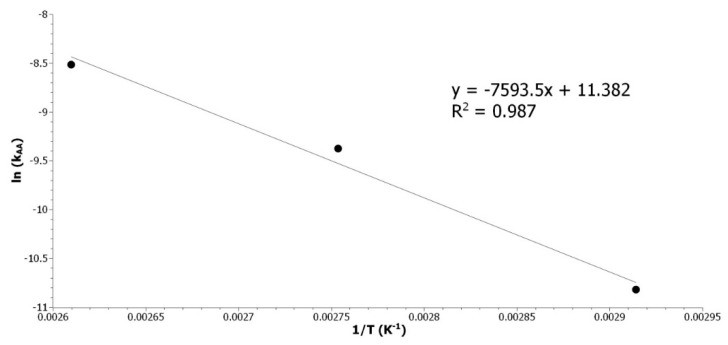
Determination of E_A_AA_ by plotting the ln(k_AA_) as a function of the inverse of the temperature.

## Supplementary Materials

The following are available online at https://www.mdpi.com/1420-3049/24/23/4332/s1. Table S1: Lipid profile. Fatty acids distribution in unsaturated FAMEVHOSO. Table S2: k_app_EtOH_ (min^−1^) with 30, 50 and 70 °C and 4, 12 and 20 wt % of amberlyst. Figure S1: NMR Spectra of the FAMEVHOSO unsaturated. Figure S2: NMR spectra of epoxidized FAMEVHOSO. Figure S3: Correlation between the mass of Amberlyst^®^ 15H and the proton quantity. Figure S4: Arrhenius law for the RO of FAMEVHOSO with ethanol at 4 (◆), 12 (▲) and 20 (●) wt% of catalyst loading. Figure S5: Representation of the gap between theoretical and experimental dosage of the NCO concentration. Figure S6: Determination of the k_aap_U_. Variation of ln([NCO]_0_/[NCO]) in function of time at 1.24 (●), 1.51 (■), 1.73 (◆), 1.98 (▲), 0.51 (▼) and 0.72 (+)mol/L. Figure S7: Application of the Arrhenius equation to determine the temperature dependency of reaction rate for the urethane formation (●) and the RO with ethanol (■). Figure S8: Conversion as a function of time for the reaction between phenyl isocyanate and VHOSO-AA (●), Hexyl isocyanate and VHOSO-AA (■) and Hexyl isocyanate and VHOSO-DEA (◆).

## Appendix A. Method Development: Kinetics of Epoxide Ring-Opening by Acetic Acid

### Appendix A.1. NMR Method for the Yield Determination

In the literature, the acetic acid rate law was determined by chemical dosage [20,21], and never by NMR. A new NMR method was developed to determine the kinetic of RO reactions. The reaction between EVHOSO and an excess of acetic acid was carried out in bulk. Aliquots were washed to remove the excess of acetic acid and then analyzed by NMR spectroscopy. The chemical shifts between the EVHOSO and the ring-opened are defined and detectable (Figure A1).

The absence of singlet at 2.10 ppm indicates the efficiency of the aliquots washing. The signals of the backbone’s protons of the methyl fatty ester were constant while the integration of the protons d adjacent to the epoxide was decreased over time. Signals of protons characteristic of the VHOSO-AA d’, d” and 1 were increased over time indicating a progression of the reaction until completion. Among the three signals indicating the progress of the reaction, the signal 1 is not specific to the RO reaction because it can be the result of transesterification. The signals of free acetic and ethyl acetate are close. The signal d” is too close to the signal of the methyl ester to be quantitative. The signal d’ was chosen to calculate the yield by comparison with the d signal, according to Equation (A1):

(A1) Yield(%)=χ=Id′1.662

where *I_d’_* is the integration of the d’ signal, 1.66 is the integration of the d signal on the EVHOSO. Based on this calculation the determination of the different partial orders and the activation energy were accomplished and compared to the literature for a validation method.

### Appendix A.2. Determination of the Epoxide Partial Order

The reaction is done without catalyst, the Equations (3) and (4) applied to the acetic acid become Equation (A2):

(A2) $$-d\frac{\left[Ep\right]}{dt}=k_{app\_AA}*{\left[Ep\right]}^{\alpha }$$

where [*Ep*] is the concentration of epoxide over time, *α* the partial order of [*Ep*]. *k_app_AA_* is detailed by Equation (A3):

(A3) $$k_{app\_AA}=k_{AA}*{\left[AA\right]}^{\beta }$$

where *k_AA_* is the rate constant of the reaction, [*AA*] the concentration of acetic acid and *β* the partial order of [*AA*].

The epoxide partial order was determined by variation of the temperature with an excess of acetic acid. The pseudo first order was applied by introducing a large excess (11 eq) of acetic acid.

The integration of the Equation (A2) with α = 1 gives Equation (A4):

(A4) $$\ln(\frac{{\left[Ep\right]}_{0}}{\left[Ep\right]})=\ln(\frac{1}{1-\chi })=k_{app\_AA}*t$$

where *χ* is the yield of the reaction and *t* the time in minute. The logarithm of [*Ep*]_0_/[*Ep*] was represented as a function of time in Figure A2. The linear regression fit the experimental data with a correlation coefficient of 0.85 for 110 °C, 0.99 for 90 and 70 °C, respectively. The lack of correlation at high temperature is the result of the fast rates of reaction and so an increased error and the side reactions.

The model of a partial order of 1 for the epoxide is well confirmed by experiments at different temperatures. The precision diminishes when the conversion is less than 20% due to the noise on the NMR spectra. The average NMR precision on the conversion is around 5% on the 20–95% interval. The precision is similar to the one obtained in previous studies [20,21].

### Appendix A.3. Determination of the Acetic Acid Partial Order

The determination of the acetic acid partial order is performed by varying the concentration of acetic acid while maintaining the great excess compared to epoxide. The acetic acid concentration was set at 8.6, 12.8 or 16.6 mol/L, by diluting with ethyl acetate. To determined k_app_AA_, the linear regression of ln([Ep]_0_/[Ep]) in function of time was plotted (Figure A3).

For the highest concentration, the coefficient of correlation is superior to 0.97. For the smallest concentration, the data points are sparser, due to the logarithm character of the model. The rate of reaction decreases at the end and reaches a plateau. In Equation A2, *k_app_AA_* depends only on [*AA*], the other factors are constant. The logarithm of Equation A2 gives the Equation (A5).

(A5) $$\ln(k_{app\_AA})=\ln(k_{AA})+\beta *\ln(\left[AA\right]),$$

The combination of Equation A5 and the *k_app_AA_* obtained at different [*AA*], linear regression of the logarithm of *k_app_AA_* as a function of the [*AA*] was presented in Figure A4, in order to determine *β*.

The partial order for [AA] was determined to be 2. The overall kinetic rate is expressed as Equation (A6):

(A6) $$r=k*\left[Ep\right]*{\left[AA\right]}^{2},$$

which is in agreement with the previous results found in the literature [20,21]. The rate constant *k* can be calculated from all the k_app_AA_ by dividing them by [*AA*]^2^. The average *k* was calculated at 70, 90 and 110 °C. The temperature dependence of *k* was modeled with an Arrhenius law described by Equation (11). The linear regression of ln(k_AA_) in function of 1/T (Figure A5) was used to determined E_A_AA_.

The energy of activation (63 kJ/mol) is in good agreement with results from the literature. The energies of activation reported are 66 kJ/mol [20] and 73 kJ/mol [21] for the RO with acetic acid of epoxidized soybean oil or epoxidized methyl ester of palm oil, respectively. The RO of disubstituted epoxide by acetic acid was studied. Despite the difference in systems and methods, the kinetic rate was found to be the same as the one in the literature and the calculated activation energy is close to the one matching our system. This is validating our NMR method for the determination of the reaction rate. Thus, it can be applied to the study of the RO of ethanol with acid catalysis.
