# Supplementary material for: New Insight on the Study of the Kinetic of Biobased Polyurethanes Synthesis Based on Oleo-Chemistry
Source: Molecules. 2019 Nov 27;24(23):4332. doi: 10.3390/molecules24234332 (PMC6935758; doi:10.3390/molecules24234332)
Supplement: Supplementary file 1 [file molecules-24-04332-s001.pdf]

# Supporting Info: New insight on the study of the kinetic of biobased polyurethanes synthesis based on oleo-chemistry

**Julien Peyrton [1], Clémence Chambaretaud [1,2] and Luc Avérous [1,*]**

[1] BioTeam/ICPEES-ECPM, UMR CNRS 7515, Université de Strasbourg, 25 rue Becquerel, 67087 Strasbourg, Cedex 2, France;
[2] Soprema, 14 rue de Saint-Nazaire, 67025 Strasbourg, Cedex 1, France;
* Correspondence: luc.averous@unistra.fr; Tel.: +333 68852784

**FAMEVHOSO Characterization:**

The repartition in fatty acid was determined according by the ISO 12966-2 method.

**Table S1.** Lipid profile. Fatty acids distribution in unsaturated FAMEVHOSO.

| **Fatty acids** | **Palmitic acid (C16:0)** | **Palmitoleoic acid (C16:1)** | **Stearic acid (C18:1)** | **Oleic acid (C18:1)** | **Acid linoleic (C18:2)** | **Linolenic acid (C18:3)** | **Arachidic acid (C20:0)** | **Eicosenoic acid (C20:1)** | **Behenic acid (C22:0)** | **Erucic acid (C22:1)** | **Other** |
|---|---|---|---|---|---|---|---|---|---|---|---|
| Repartition (%) | 3.58 | 0.12 | 2.71 | 82.78 | 9.07 | 0.06 | 0.25 | 0.21 | 0.84 | 0.29 | 0.09 |

The average double bond per molecule is determined from the number of double bond per fatty acid.

The NMR spectra of the FAMEVHOSO and EVHOSO are presented on Figure S1 and Figure S2, respectively.

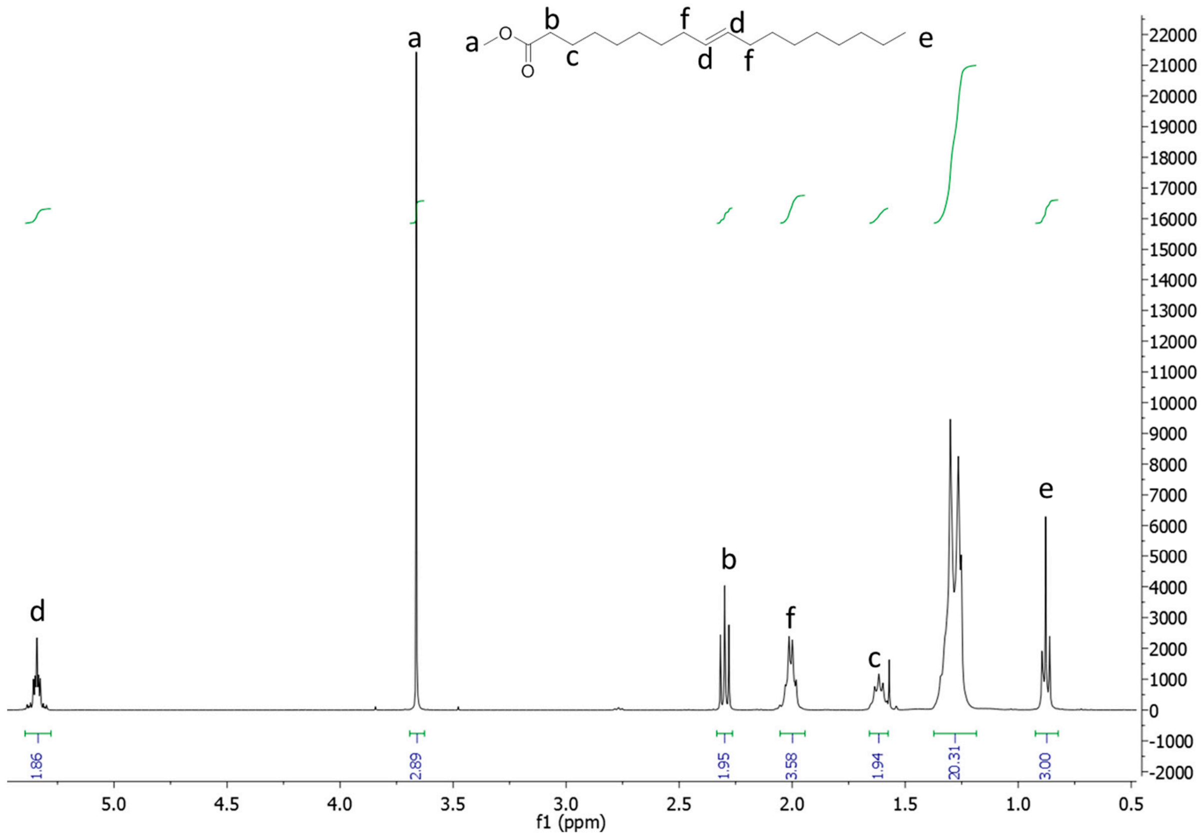


**Figure S1.** NMR Spectra of the FAMEVHOSO unsaturated.

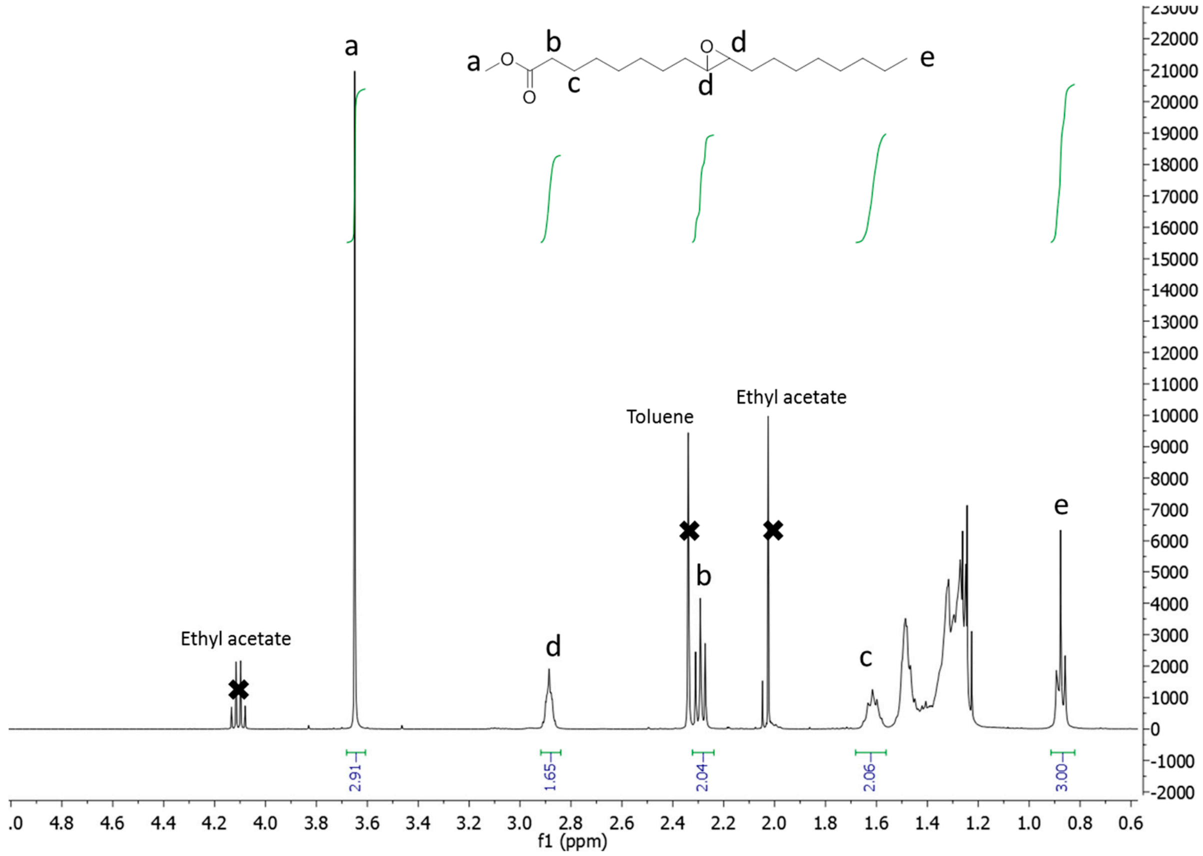


**Figure S2.** NMR spectra of epoxidized FAMEVHOSO.

**Variation of kapp_EtOH with different temperature and catalyst quantity:**

By combining the linear regression slope of experiment performed at 30, 50 and 70 °C with 4, 12 and 20 wt% of amberlyst, respectively, Table S2 is obtained.

**Table S2.** $k_{app\_EtOH}$ (min$^{-1}$) with 30, 50 and 70 °C and 4, 12 and 20 wt % of amberlyst.

| Amberlyst wt% eq | k' (30) | k' (50) | k' (70) |
|---|---|---|---|
| 4 | 0.000601 | 0.00241 | 0.0075 |
| 12 | 0.00114 | 0.00439 | 0.0137 |
| 20 | 0.00119 | 0.0071 | 0.0141 |

## Correlation between the mass of catalyst and the quantity of $H^+$:

In order to know the relation between the mass of catalyst and the proton quantity, an experiment was designed: Different mass of Amberlyst® 15H was put in a beaker with 40 mL of water, then the pH was measured. Experimental results are shown in Figure S3.

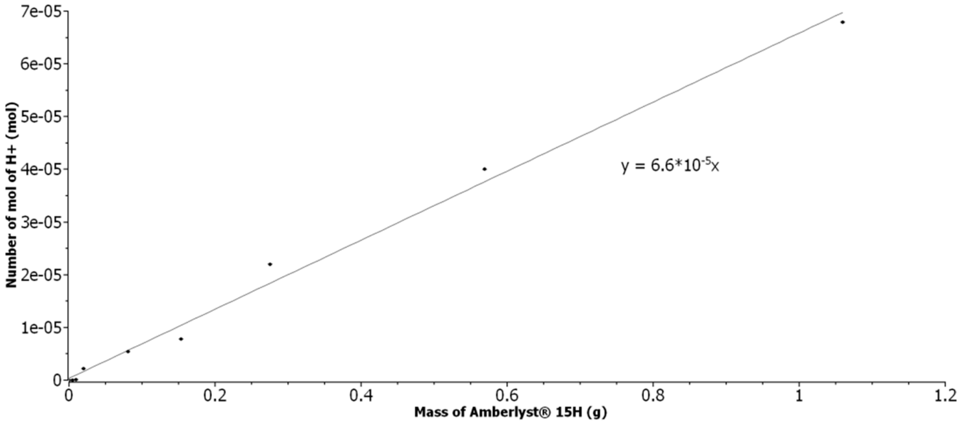


**Figure S3.** Correlation between the mass of Amberlyst® 15H and the proton quantity.

## Determination of the Activation energy of RO with ethanol:

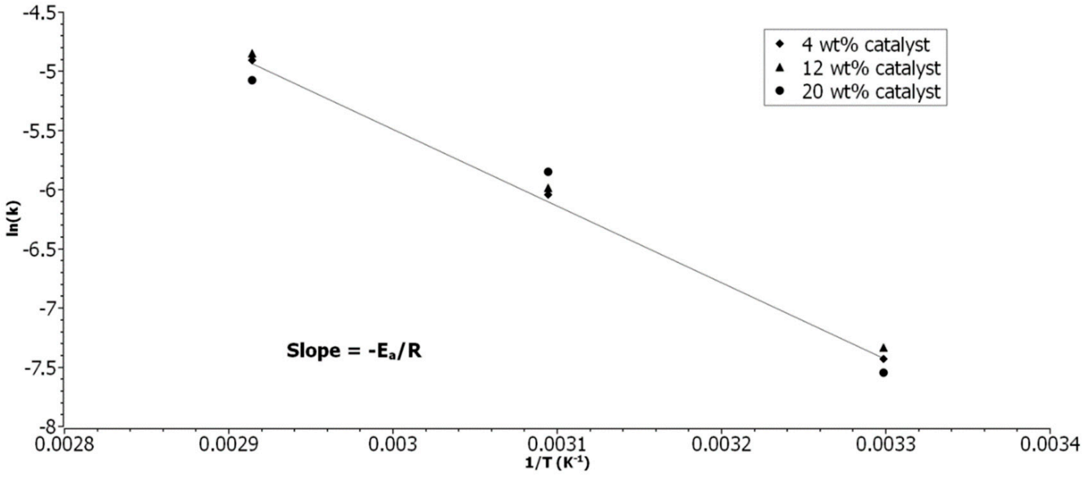


**Figure S4.** Arrhenius law for the RO of FAMEVHOSO with ethanol at 4 (◆), 12 (▲) and 20 (●) wt% of catalyst loading.

## Method validation for the NCO titration:

A known concentration of phenyl isocyanate was titrated by the protocol. The value obtained was then compared with the theoretical one in Figure S5.

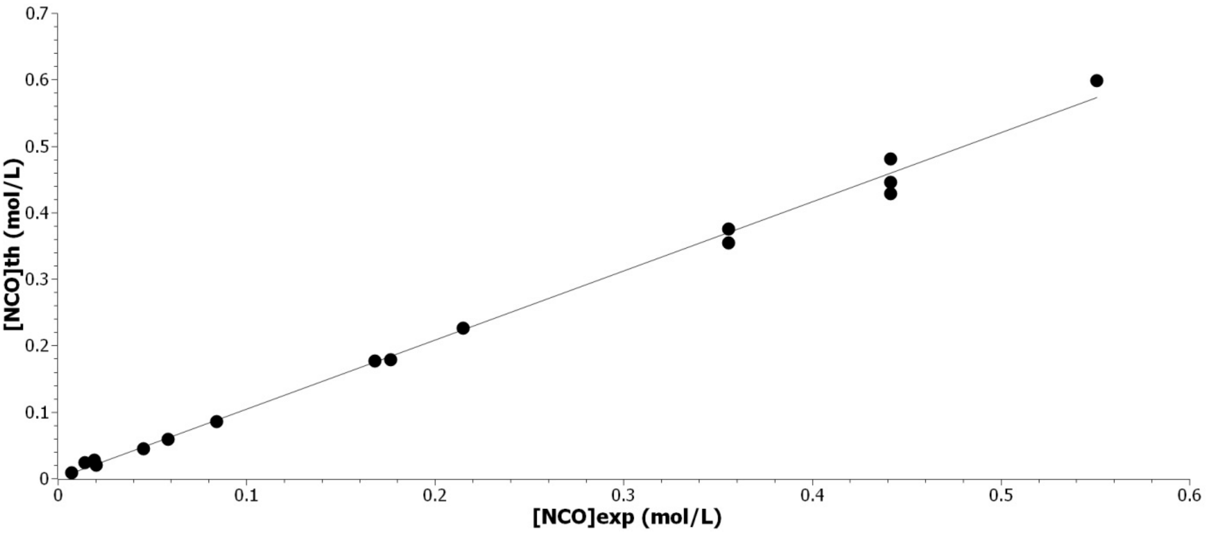


**Figure S5.** Representation of the gap between theoretical and experimental dosage of the NCO concentration.

The linear regression slope is 1.04. The error made on the dosage is 4 % between 7*10-3 and 0.6 mol/L of isocyanate groups.

**Determination of $k_{aap\_U}$ with hydroxyl concentration variation:**

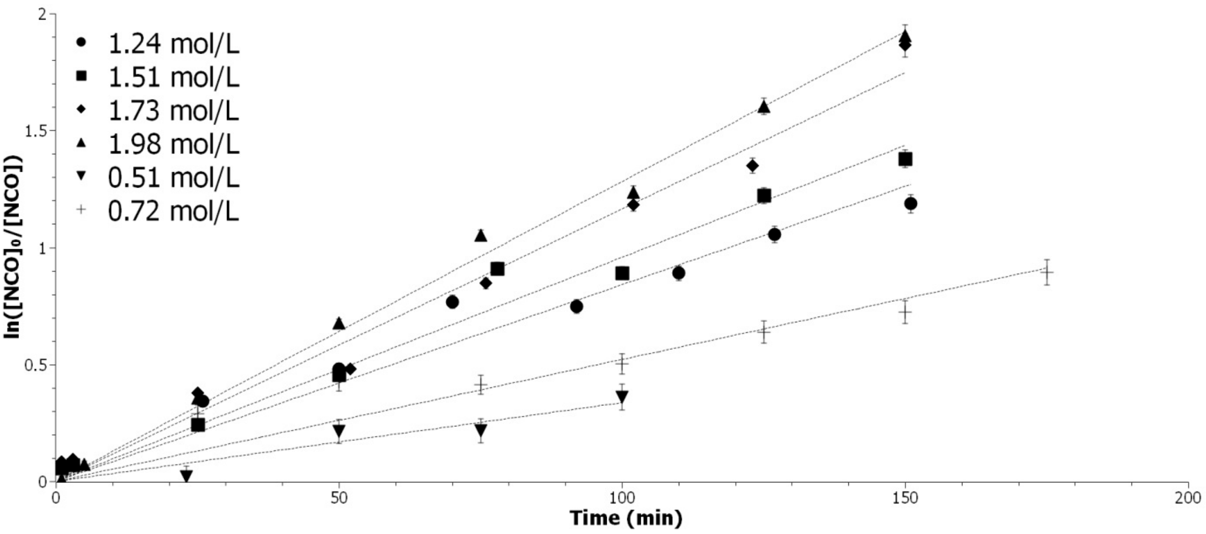


**Figure S6.** Determination of the kaap_U. Variation of ln([NCO]0/[NCO]) in function of time at 1.24 (●), 1.51 (■), 1.73 (◆), 1.98 (▲), 0.51 (▼) and 0.72 mol/L(+ ).

**Comparison between urethane formation and RO with ethanol:**

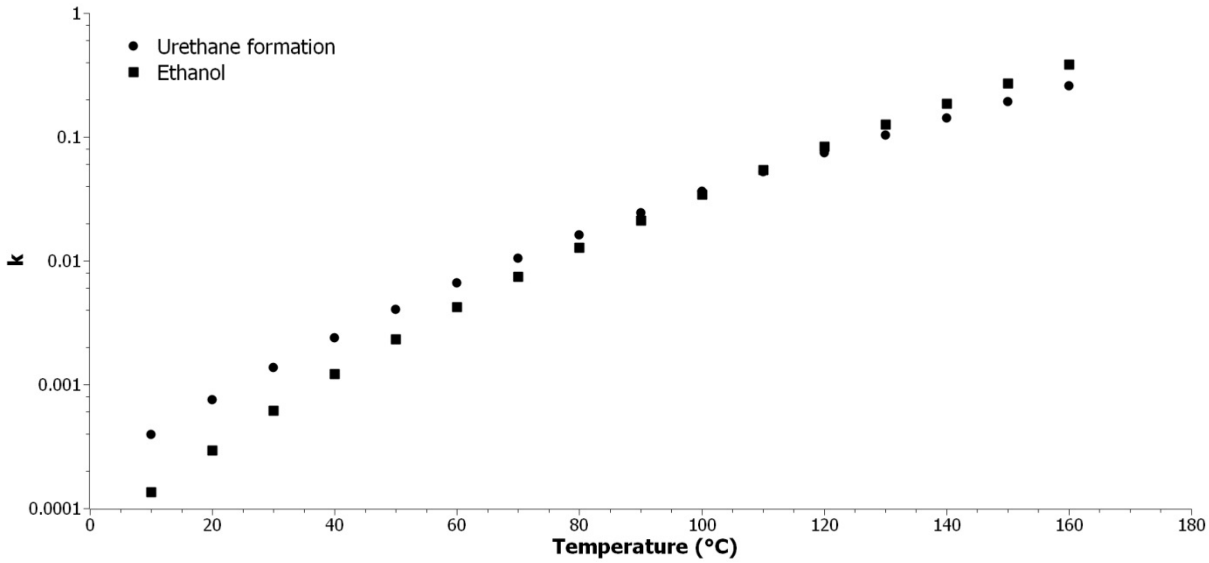


**Figure S7.** Application of the Arrhenius equation to determine the temperature dependency of reaction rate for the urethane formation (●) and the RO with ethanol (■).

## Reaction of VHOSO-DEA with aliphatic and aromatic isocyanate:

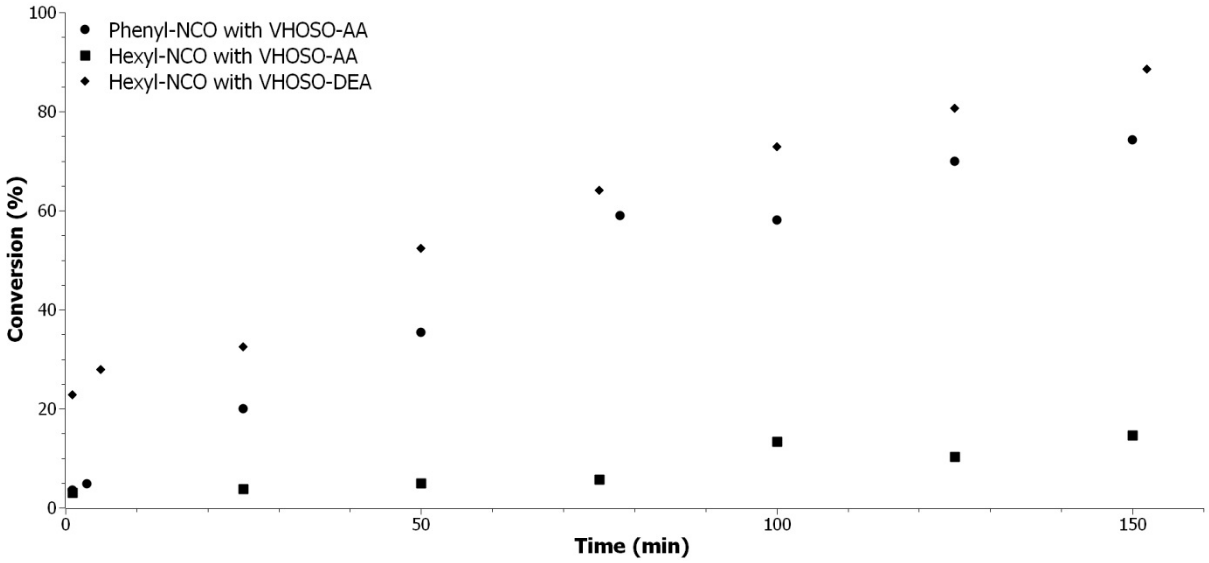


**Figure S8.** Conversion as a function of time for the reaction between phenyl isocyanate and VHOSO-AA (●), Hexyl isocyanate and VHOSO-AA (■) and Hexyl isocyanate and VHOSO-DEA (◆ ).
